# Sociodemographic and Health Characteristics Associated With Attempting Weight Loss During Pregnancy

**Published:** 2008-12-15

**Authors:** Jennifer H. Cohen, Hyoshin Kim

**Affiliations:** Battelle Centers for Public Health Research and Evaluation; Battelle Centers for Public Health Research and Evaluation, Seattle, Washington

## Abstract

**Introduction:**

Approximately 40% of women of childbearing age report that they are attempting to lose weight. No professional medical organization recommends attempting to lose weight during pregnancy because of the possible risks to both mother and baby. Since half of all pregnancies are unintended, women may attempt to lose weight before they know they are pregnant, and some women may continue or initiate weight loss attempts even after they know they are pregnant. This study examines the extent to which pregnant women report attempting to lose weight and associated sociodemographic and health characteristics.

**Methods:**

We used aggregated multiple-year data (1996-2003) from the Behavioral Risk Factor Surveillance System to assess the prevalence of attempting to lose weight among pregnant women and the extent to which sociodemographic and health characteristics are associated with the behavior.

**Results:**

The prevalence of attempting to lose weight during pregnancy was 8.1%. Attempting to lose weight during pregnancy was associated with age 35-44 years, Hispanic ethnicity, obesity, alcohol consumption, and mental distress during the previous month.

**Conclusion:**

A substantial proportion of pregnant women attempt to lose weight. Preconception and prenatal care should include counseling women to achieve a healthy weight before becoming pregnant, to maintain healthy weight during pregnancy, and not to attempt weight loss during pregnancy. Further research should be conducted to understand how attempting weight loss during pregnancy translates into dietary change and weight loss and associated maternal and fetal outcomes.

## Introduction

Approximately 40% of women of childbearing age report that they are attempting to lose weight (1). Because half of all pregnancies are unintended (2), pregnant women may attempt to lose weight before they know they are pregnant. Most women believe that weight gain in pregnancy is positive and cease weight loss efforts when they realize they are pregnant (3), and no professional medical organization or public health agency recommends attempting to lose weight during pregnancy because of the possible risks to both the mother (complications at childbirth) and baby (neural tube defects [NTDs], preterm delivery, increased lifetime risk of diabetes) (4-13), even for obese women. Nevertheless, some women may continue or initiate weight loss attempts even after they know they are pregnant because of negative perceptions of weight gain and dissatisfaction with body weight (14-16).

Attempting to lose weight during pregnancy is problematic only if it leads to changes in diet that impair maternal and fetal health. Attempting to lose weight is associated with low caloric and micronutrient intake (9,17-19), but few studies have examined outcomes associated with attempting weight loss during pregnancy. In a population-based, case-control study, attempting to lose weight was associated with an increased risk of NTDs in the infant (9). Among women who reported attempting to lose weight during the first trimester of pregnancy, the odds of NTD were 2.1 (95% confidence interval [CI], 1.1-4.1) higher than among women who did not report attempting to lose weight (9). Another recent study suggests that weight loss among obese pregnant women may reduce the risks for preeclampsia, cesarean delivery, small-for-gestational-age birth, and large-for-gestational-age birth; however, risk levels for the 4 outcomes did not vary between women who did not gain any weight and women who lost weight across all categories of obesity (20). Furthermore, the study was restricted to full-term live births, which may have excluded adverse maternal and fetal outcomes associated with maternal weight loss, and the study did not include women who were not obese.

Other studies of correlates of attempting weight loss during pregnancy are limited by insufficient samples of pregnant women (21) or are limited in generalizability by study designs, such as case series or clinic-based, or tend to focus on pathologic eating disorders or fasting (14,22-25). The most recent estimate of the extent to which pregnant women attempt to lose weight is based on data from 16 years ago (21). Despite the lack of evidence on maternal and fetal outcomes associated with attempted weight loss and actual weight loss during pregnancy, the behavior is not recommended and may indicate, like smoking and alcohol consumption, a high-risk pregnancy. Understanding the prevalence of attempting weight loss during pregnancy and factors associated with this potentially unhealthful behavior will inform future outcomes research and aid in developing appropriate interventions to prevent and control the behavior.

## Methods

We aggregated data from the Behavioral Risk Factor Surveillance System (BRFSS) from 1996, 1998, 2000, and 2003 — the 4 most recent years that included the weight-loss module as part of the core survey. This method provided sufficient numbers of pregnant women to evaluate sociodemographic and health correlates of attempting to lose weight. Data from interim years are not included because weight-loss measurements were not part of the core BRFSS survey instrument. Pooling multiple years of BRFSS data can help estimate prevalence of health behaviors and diseases, and their correlates, among pregnant women (26-28).

BRFSS is an ongoing, state-based, random-digit–dialed telephone survey of the noninstitutionalized US population aged 18 years or older in all 50 states, the District of Columbia, and US territories. BRFSS includes a core survey with questions that are asked each year by all states, rotating questions asked in alternating years by all states, optional modules that states may elect to ask, and questions that are added by individual states. Median response rates varied by state for the 4 years of data, from approximately 50% to 65% (29,30). Rates of missing data are minimal for pregnancy status and attempting weight loss across all years of the survey. BRFSS has higher rates of missing data for alcohol consumption, income, and mental health distress. A detailed description of the BRFSS survey methods and data are available on the BRFSS Web site (www.cdc.gov/brfss).

Our sample consisted of 8,036 pregnant women aged 18 to 44 who had information on attempting to lose weight. Missing data rates for other main variables of interest, including demographic (race and ethnicity, age, marital status, and number of children younger than 18 in the household), socioeconomic (education and household income), and health (health care coverage, mental health, smoking, diabetes, and body mass index) characteristics, are consistent with BRFSS analyses of data for women and men and with what is seen in other survey research for similar variables. Percentages of missing data range from less than 0.01% for demographic variables to 10% for income. The sample that was available for multivariate analysis was 6,593 pregnant women with data on all variables of interest. Because data on alcohol consumption were collected only in selected states, a subsample of 3,315 pregnant women who provided data on alcohol consumption was used for the multivariate model that included alcohol use and binge drinking as covariates.

Pregnancy status was self-reported. Women were asked, "To your knowledge, are you now pregnant?" Those who answered yes were included for analysis as pregnant women. In a separate module of the survey, those respondents were also asked, "Are you now trying to lose weight?" Those who answered yes were defined for the analysis as attempting to lose weight.

The following demographic characteristics were constructed for analyses: 4 categories of race/ethnicity (white, black, Hispanic, or other), 4 categories of age (18-24, 25-29, 30-34, or 35-44 years), 4 categories of marital status (married; divorced, separated, or widowed; never married; or member of an unmarried couple), and 2 categories for number of children younger than 18 in the household (0 or ≥1). Our 3 socioeconomic measures included 4 categories of education (less than high school graduate, high school graduate, some college, or college graduate), 5 categories of household income (<$15,000, $15,000-$24,999, $25,000-$49,999, $50,000-$74,999, or ≥$75,000), and 2 categories of health care coverage (having or not having coverage). Coverage was based on the question, "Do you have any kind of health care coverage, including health insurance, prepaid plans such as a health maintenance organization, or government plans such as Medicare?"

We also constructed measures for 3 health-related characteristics. Three categories of mental distress were based on the response to the question, "For how many days during the past 30 days was your mental health not good?" (0, ≤13, or >13). The measure of diabetes was based on the question, "Has your doctor ever told you that you have diabetes, including during pregnancy?" Respondents who reported currently smoking were categorized as currently smoking. On the basis of self-reported body weight and height, we calculated 4 categories of body mass index (BMI): underweight (BMI <18.5 kg/m^2^), normal weight (BMI 18.5-24.9 kg/m^2^), overweight (BMI 25.0-29.9 kg/m^2^), or obese (BMI ≥30.0 kg/m^2^).

We used the subsample of 3,315 pregnant women who had information on alcohol consumption to explore the associations of alcohol consumption and binge drinking with attempting to lose weight. Alcohol consumption was defined as having had at least 1 drink in the past month, and binge drinking was defined as having had at least 5 drinks on 1 occasion in the past month.

We estimated overall prevalence of attempting to lose weight among pregnant women and the prevalences by sociodemographic and health characteristics. Unadjusted odds ratios of attempting to lose weight among pregnant women based on sociodemographic and health characteristics were estimated from logistic regression models. Adjusted odds ratios were also estimated in multivariate models that controlled for sociodemographic and health characteristics. To account for the complex sampling design of BRFSS, we used Stata version 9 (StataCorp LP, College Station, Texas) survey procedures for all of the analyses.

## Results

The proportion of pregnant women who were attempting to lose weight was 8.1% (95% CI, 7.0%-9.2%). This proportion did not vary significantly across years of data. Most pregnant women were younger than 35, white, married, and lived in a household with 1 or more children younger than 18 years (Table 1). Approximately 80% of the sample had at least a high school education, and one-third lived in a household with an income of $50,000 or more per year. Most women reported having health insurance coverage. For the subanalysis, 10.7% (95% CI, 9.1%-12.3%) women reported having had at least 1 drink in the past month, and approximately 1.7% (95%, CI 1.1%-2.3%) reported binge drinking in the past month.

The ratio of pregnant women who were attempting to lose weight was highest among women older than 34 (Table 2). Hispanic women were more likely to attempt to lose weight than were white women. Married women were less likely to attempt to lose weight than were women who were divorced, separated, widowed, or never married. Women with 1 or more children at home were more likely to attempt to lose weight than were women without children at home. The proportion of women who were attempting to lose weight generally increased as level of education decreased. Women who lived in households with an annual income of less than $15,000 were more likely to attempt to lose weight than were women with household income greater than $75,000. Women who had no health insurance, who were currently smoking, or who had diabetes were more likely to attempt to lose weight than their counterparts. Women who experienced any mental distress in the past month were more likely to attempt to lose weight than were women who experienced no mental distress. Obese women were more likely to attempt to lose weight than were women of normal weight. In the alcohol subanalysis, binge drinkers were significantly more likely to attempt to lose weight than nonbinge drinkers, and women who reported consuming at least 1 drink in the past month were more likely to report attempting to lose weight than nondrinkers.

After adjusting for covariates, the associations of most variables with attempting to lose weight during pregnancy were attenuated, and some of the variables became nonsignificant. Age 35 years or older, Hispanic ethnicity, having 1 or more children at home, reporting any days of mental distress in the past 30 days, and BMI of 30 or higher remained significantly associated with attempting to lose weight during pregnancy after adjusting for covariates. For the alcohol subanalysis, after adjusting for covariates, the odds of attempting weight loss were higher for drinkers than for nondrinkers, and the association with binge drinking became nonsignificant. Slight changes in the odds ratios for some covariates were observed, which suggests that alcohol use during pregnancy may be associated with sociodemographic characteristics and attempting weight loss.

## Discussion

We found that a substantial proportion (8.1%) of pregnant women report attempting to lose weight. Attempting to lose weight during pregnancy is associated with being older than 34, Hispanic ethnicity, obesity, alcohol consumption, and mental distress in the past month. This figure may be an underestimate of the true prevalence because women may attempt to lose weight before they learn that they are pregnant and because pregnant women may underreport the behavior if they believe that it is socially undesirable.

Our findings are consistent with those of other studies that found that women who experience mental distress are more likely to attempt to lose weight or engage in high-risk dietary or other health behaviors (15,19,25-27,29-31) and with studies that show that women of childbearing age who are overweight or obese or older than 34 are more likely to attempt to lose weight (1,21). The magnitude of the association between Hispanic ethnicity and attempting to lose weight, while significant, was small and disappeared when alcohol use was accounted for. The subsample analysis that included alcohol use shows that alcohol consumption in the past month is associated with attempting to lose weight in pregnant women. This finding may indicate that certain high-risk behaviors coexist among pregnant women.

This study has several limitations that must be considered in its interpretation. First, BRFSS is a telephone-based survey that does not reach people without telephones or who only have cell phones. It is also a cross-sectional study, which limits conclusions regarding causal associations. Second, BMI data are based on self-report. Respondents tend to overreport height and underreport weight; however, we found an association between obesity and attempting to lose weight. Third, no measure of week of gestation was available in the BRFSS data. Although measures of mental health status and alcohol consumption represent behavior during the past month and may indicate prepregnancy behavior in women who have been pregnant for less than a month, most women do not know they are pregnant until at least a month after conception. A previous study (21) reported that most women who were attempting to lose weight were in the first trimester of pregnancy. Without information on gestational stage, we do not know if the prevalence of attempting weight loss varies by gestational stage or if the observed associations vary by gestational stage. Fourth, we did not have information to evaluate the intensity or effectiveness of weight-loss attempts or what specific methods women may have used to lose weight. Different methods — such as exercise, caloric restriction, increased protein intake, lower fat intake, herbal supplements, binge eating, vomiting, diet pills, laxatives, water pills, and skipping meals — may be associated with better or worse outcomes (1,32).

The health consequences of obesity (including complications of pregnancy) are well documented (4,33), and the health benefits of even small amounts of prepregnancy weight loss are evident (4). Clinicians and public health agencies do not recommend attempting weight loss during pregnancy (4,8,34); however, a substantial proportion of pregnant women report attempting to lose weight. Further research should be conducted to understand the extent to which attempting to lose weight translates into dietary change and weight loss and associated maternal and fetal outcomes. Given the high prevalence of attempting to lose weight during pregnancy and the association with other high-risk behaviors, such as alcohol consumption and mental health distress, preconceptional and prenatal care should include counseling women to achieve a healthy weight before becoming pregnant, to maintain a healthy weight during pregnancy, and not to attempt weight loss during pregnancy.

## Figures and Tables

**Table 1:** Characteristics of Pregnant Women Who Were Attempting to Lose Weight, Behavioral Risk Factor Surveillance System (1996, 1998, 2000, 2003), United States

**Characteristic**	No. of Respondents	Weighted % (SE)^a^
**Age, y**		
18-24	2,225	31.8 (0.9)
25-29	2,469	28.6 (0.8)
30-34	2,167	25.3 (0.8)
35-44	1,286	14.3 (0.6)
**Race/ethnicity**		
White	5,787	64.9 (0.9)
Black	749	10.2 (0.6)
Hispanic	1,047	19.5 (0.8)
Other	544	5.4 (0.4)
**Marital status**		
Married	5,943	71.9 (0.9)
Divorced, separated, widowed	527	5.5 (0.4)
Never married	1,222	16.5 (0.7)
Member of unmarried couple	447	6.2 (0.5)
**No. of children aged <18 y at home**		
0	2,654	33.9 (0.9)
≥1	5,483	66.1 (0.9)
**Household income, $**		
<15,000	831	13.0 (0.7)
15,000-24,999	1,383	20.3 (0.8)
25,000-49,999	2,537	34.1 (0.9)
50,000-74,999	1,278	16.0 (0.7)
≥75,000	1,158	16.7 (0.7)
**Education level**		
Less than high school graduate	804	14.5 (0.8)
High school graduate	2,212	23.4 (0.8)
Some college	2,342	26.8 (0.8)
College graduate	2,783	30.2 (0.8)
**Health insurance coverage**		
Yes	7,327	87.6 (0.7)
No	813	12.4 (0.7)
**Mental health status**		
No mental health distress (0 days in past 30 days)	5,172	64.1 (0.9)
Few days of mental health distress (1-13 days in past 30 days)	2,246	28.8 (0.8)
Frequent mental health distress (≥14 days in past 30 days)	619	7.1 (0.4)
**Smoking**		
Not current	7,136	88.2 (0.6)
Current	999	11.8 (0.6)
**Alcohol consumption**		
No alcohol consumption	3,692	89.3 (0.8)
≥1 Drink in past month	462	10.7 (0.8)
**Binge drinking**		
No binge drinking	4,073	98.3 (0.3)
≥5 Drinks on 1 occasion in past month	78	1.7 (0.3)
**Ever told by doctor that you have diabetes, including during pregnancy**		
No	7,804	96.6 (0.3)
Yes	339	3.4 (0.3)
**Body mass index, kg/m^2^**		
<18.5 (Underweight)	183	2.5 (0.3)
18.5-24.9 (Normal weight)	3,682	47.4 (0.9)
25.0-29.9 (Overweight)	2,271	29.9 (0.9)
≥30.0 (Obese)	1,467	20.2 (0.8)

Abbreviation: SE, standard error.

a Percentages may not add to 100% because of rounding.

**Table 2:** Associations Between Sociodemographic and Health Characteristics and Attempting to Lose Weight Among Pregnant Women, Behavioral Risk Factor Surveillance System (1996, 1998, 2000, 2003)

Characteristic	% of Women Attempting to Lose Weight (95% CI)	Unadjusted OR (95% CI)	Adjusted OR (95% CI)^a^	

			Full Sample (N = 6,593)	Restricted Subsample^b^ of Alcohol Users (n = 3,315)
**Age, y**				
18-24	7.1 (5.4-9.2)	1 [Referent]	1 [Referent]	1 [Referent]
25-29	8.1 (6.3-10.3)	1.2 (0.8-1.7)	1.4 (0.9-2.4)	1.6 (0.7-3.6)
30-34	6.2 (4.8-7.9)	0.9 (0.6-1.3)	1.2 (0.7-2.0)	1.5 (0.8-3.0)
35-44	13.6 (10.6-17.2)	2.1 (1.4-3.1)	2.7 (1.5-4.7)	2.9 (1.3-6.6)
**Race/ethnicity**				
White	6.5 (5.5-7.6)	1 [Referent]	1 [Referent]	1 [Referent]
Black	8.0 (4.9-12.8)	1.3 (0.7-2.2)	1.1 (0.5-2.2)	1.0 (0.5-2.3)
Hispanic	13.1 (10.2-16.7)	2.2 (1.6-3.0)	1.7 (1.1-2.7)	1.0 (0.5-2.2)
Other	8.5 (5.1-14.0)	1.3 (0.8-2.4)	1.6 (0.8-3.1)	1.4 (0.6-3.0)
**Marital status**				
Married	7.0 (5.9-8.2)	1 [Referent]	1 [Referent]	1 [Referent]
Divorced, separated, widowed	13.6 (9.0-20.0)	2.1 (1.3-3.4)	1.2 (0.7-2.0)	0.5 (0.2-1.4)
Never married	11.1 (8.4-14.4)	1.7 (1.2-2.3)	1.5 (0.8-2.7)	2.0 (0.9-4.4)
Member of unmarried couple	8.2 (4.7-14.1)	1.2 (0.6-2.2)	0.9 (0.4-2.1)	0.8 (0.2-3.9)
**No. of children aged <18 y at home**				
0	5.9 (4.6-7.5)	1 [Referent]	1 [Referent]	1 [Referent]
≥1	9.2 (7.9-10.7)	1.6 (1.2-2.2)	1.5 (1.1-2.2)	1.7 (1.0-3.1)
**Household income, $**				
<15,000	13.3 (9.8-18.0)	3.0 (1.8-5.0)	1.6 (0.8-3.1)	2.0 (0.6-6.4)
15,000-24,999	7.3 (5.3-10.0)	1.6 (0.9-2.6)	1.0 (0.5-1.8)	1.2 (0.4-3.0)
25,000-49,999	8.2 (6.3-10.6)	1.8 (1.1-2.8)	1.5 (0.9-2.4)	2.0 (0.9-4.2)
50,000-74,999	6.7 (5.0-9.1)	1.4 (0.9-2.3)	1.4 (0.8-2.4)	3.3 (1.5-7.0)
≥75,000	4.8 (3.3-6.8)	1 [Referent]	1 [Referent]	1 [Referent]
**Education level**				
Less than high school graduate	11.0 (7.8-15.3)	2.1 (1.3-3.2)	1.2 (0.6-2.2)	2.3 (0.8-6.7)
High school graduate	8.7 (6.9-11.0)	1.6 (1.1-2.3)	1.3 (0.8-2.0)	2.0 (1.1-3.6)
Some college	8.6 (6.8-10.8)	1.6 (1.1-2.2)	1.2 (0.8-1.8)	1.7 (0.9-3.0)
College graduate	5.6 (4.5-7.0)	1 [Referent]	1 [Referent]	1 [Referent]
**Health insurance coverage**				
Yes	7.5 (6.5-8.7)	1 [Referent]	1 [Referent]	1 [Referent]
No	11.9 (8.8-16.0)	1.7 (1.1-2.4)	1.5 (0.9-2.4)	1.4 (0.5-3.4)
**Mental health status**				
No mental health distress (0 days in past 30 days)	6.46 (5.4-7.5)	1 [Referent]	1 [Referent]	1 [Referent]
Few days of mental health distress (1-1-13 days in past 30 days)	10.4 (8.1-13.2)	1.7 (1.2-2.3)	1.7 (1.2-2.4)	2.0 (1.1-3.4)
Frequent mental health distress (≥14 days in past 30 days)	14.6 (10.6-19.7)	2.5 (1.7-3.8)	2.2 (1.4-3.6)	1.7 (0.8-3.7)
**Smoking**				
Not current	7.3 (6.3-8.6)	1 [Referent]	1 [Referent]	1 [Referent]
Current	12.3 (9.4-15.9)	1.7 (1.2-2.4)	1.3 (0.8-2.0)	1.1 (0.5-2.2)
**Alcohol consumption**				
No alcohol consumption	5.5 (4.2-7.2)	1 [Referent]	NA	1 [Referent]
≥1 Drink in past month	19.7 (14.6-26.0)	4.2 (2.7-6.6)	NA	3.7 (2.0-6.9)
**Binge drinking**				
No binge drinking	6.6 (5.3-8.2)	1 [Referent]	NA	1 [Referent]
≥5 Drinks on 1 occasion in past month	32.0 (18.6-49.2)	6.7 (3.1-14.3)	NA	2.0 (0.7-6.2)
**Ever told by doctor that you have diabetes, including during pregnancy**				
No	7.9 (6.9-9.0)	1 [Referent]	1 [Referent]	1 [Referent]
Yes	14.6 (9.5-21.9)	2.0 (1.2-3.4)	1.5 (0.9-2.7)	2.1 (0.9-4.9)
**Body mass index, kg/m^2^**				
<18.5 (Underweight)	2.9 (1.1-7.5)	0.4 (0.2-1.2)	0.2 (0.1-0.9)	0.3 (0.03-2.6)
18.5-24.9 (Normal weight)	6.7 (5.3-8.3)	1 [Referent]	1 [Referent]	1 [Referent]
25.0-29.9 (Overweight)	7.9 (6.3-9.9)	1.2 (0.9-1.7)	1.2 (0.8-1.7)	1.2 (0.6-2.3)
≥30.0 (Obese)	12.3 (9.6-15.6)	2.0 (1.4-2.8)	1.8 (1.2-2.5)	1.8 (1.0-3.4)

Abbreviations: CI, confidence interval; OR, odds ratio; NA, not applicable.

a Indicators for different survey years were included to control for variations across survey years in the multivariate models.

b Information on alcohol consumption was not available from the core Behavioral Risk Factor Surveillance System survey in each of the 4 years, which reduced the sample size for analysis to 3,315 pregnant women.

## References

[1] Kruger J, Galuska DA, Serdula MK, Jones DA (2004). Attempting to lose weight: specific practices among US adults. Am J Prev Med.

[2] Henshaw SK (1998). Unintended pregnancy in the United States. Fam Plann Perspect.

[3] Rocco PL, Orbitello B, Perini L, Pera V, Ciano RP, Balestrieri M (2005). Effects of pregnancy on eating attitudes and disorders: a prospective study. J Psychosom Res.

[4] Johnson K, Posner SF, Biermann J, Cordero JF, Atrash HK, Parker CS (2014). Recommendations to improve preconception health and health care — United States. A report of the CDC/ATSDR Preconception Care Work Group and the Select Panel on Preconception Care. MMWR Recomm Rep.

[5] Dewey KG, McCrory MA (1994). Effects of dieting and physical activity on pregnancy and lactation. Am J Clin Nutr.

[6] Anderson AS (2001). Symposium on nutritional adaptation to pregnancy and lactation. Pregnancy as a time for dietary change?. Proc Nutr Soc.

[7] Kaiser LL, Allen L (2002). Position of the American Dietetic Association: nutrition and lifestyle for a healthy pregnancy outcome. J Am Diet Assoc.

[8] (2005). American College of Obstetricians and Gynecologists. ACOG Committee Opinion number 315, September 2005. Obesity in pregnancy. Obstet Gynecol.

[9] Carmichael SL, Shaw GM, Schaffer DM, Laurent C, Selvin S (2003). Dieting behaviors and risk of neural tube defects. Am J Epidemiol.

[10] Shaw GM, Todoroff K, Carmichael SL, Schaffer DM, Selvin S (2001). Lowered weight gain during pregnancy and risk of neural tube defects among offspring. Int J Epidemiol.

[11] Siega-Riz AM, Herrmann T, Savitz DA, Thorp J (2001). The frequency of eating during pregnancy and its effect on preterm delivery. Am J Epidemiol.

[12] Herrmann TS, Siega-Riz AM, Hobel CJ, Aurora C, Dunkel-Schetter C (2001). Prolonged periods without food intake during pregnancy increase risk for elevated maternal corticotropin-releasing hormone concentrations. Am J Obstet Gynecol.

[13] Stocker CJ, Arch JR, Cawthorne MA (2005). Fetal origins of insulin resistance and obesity. Proc Nutr Soc.

[14] Franko DL, Blais MA, Becker AE, Delinsky SS, Greenwood DN, Flores AT (2001). Pregnancy complications and neonatal outcomes in women with eating disorders. Am J Psychiatr.

[15] Dipietro JA, Millet S, Costigan KA, Gurewitsch E, Caulfield LE (2003). Psychosocial influences on weight gain attitudes and behaviors during pregnancy. J Am Diet Assoc.

[16] Hickey CA (2000). Sociocultural and behavioral influences on weight gain during pregnancy. Am J Clin Nutr.

[17] Neumark-Sztainer D, French SA, Jeffery RW (1996). Dieting for weight loss: associations with nutrient intake among women. J Am Diet Assoc.

[18] Kant AK (2002). Weight-loss attempts and reporting of foods and nutrients, and biomarkers in a national cohort. Int J Obes Relat Metab Disord.

[19] Laessle RG, Tuschl RJ, Kotthaus BC, Pirke KM (1989). Behavioral and biological correlates of dietary restraint in normal life. Appetite.

[20] Kiel DW, Dodson E, Artal R, Boehmer TK, Leet TL (2007). Gestational weight gain and pregnancy outcomes in obese women: how much is enough?. Obstet Gynecol.

[21] Cogswell ME, Serdula MK, Mokdad AH, Williamson DF (1996). Attempted weight loss during pregnancy. Int J Obes Relat Metab Disord.

[22] Park RJ, Senior R, Stein A (2003). The offspring of mothers with eating disorders. Eur Child Adolesc Psychiatry.

[23] Pearlstein T (2002). Eating disorders and comorbidity. Arch Womens Ment Health.

[24] Turton P, Hughes P, Bolton H, Sedgwick P (1999). Incidence and demographic correlates of eating disorder symptoms in a pregnant population. Int J Eat Disord.

[25] Mirghani HM, Hamud OA (2006). The effect of maternal diet restriction on pregnancy outcome. Am J Perinatol.

[26] Ahluwalia IB, Mack KA, Mokdad A (2004). Mental and physical distress and high-risk behaviors among reproductive-age women. Obstet Gynecol.

[27] Petersen AM, Leet TL, Brownson RC (2005). Correlates of physical activity among pregnant women in the United States. Med Sci Sports Exerc.

[28] Iachan R, Schulman J, Collins S, Powell-Griner E, Nelson D (1999). Methods for combining state BRFSS survey data for national estimation. Proceedings of the Section on Survey Research Methods, American Statistical Association.

[29] 2000 BRFSS summary data quality report..

[30] 2003 BRFSS summary data quality report.

[31] Cohen JH, Kristal AR, Neumark-Sztainer D, Rock CL, Neuhouser ML (2002). Psychological distress is associated with unhealthful dietary practices. J Am Diet Assoc.

[32] Hurley KM, Caulfield LE, Sacco LM, Costigan KA, Dipietro JA (2005). Psychosocial influences in dietary patterns during pregnancy. J Am Diet Assoc.

[33] Galtier-Dereure F, Boegner C, Bringer J (2000). Obesity and pregnancy: complications and cost. Am J Clin Nutr.

[34] (1990). Institute of Medicine. Nutrition during pregnancy.

